# Self-reported health as a predictor of cardiometabolic multimorbidity in Chinese older adults: a national cross-sectional study

**DOI:** 10.3389/fpubh.2025.1691960

**Published:** 2025-11-14

**Authors:** Siyu Bing, Weida Liu, Aihong Wang, Shuwen Mao, Xiaoyun Teng, Qiaoqiao Wang

**Affiliations:** 1 School of Nursing, Shandong Second Medical University, Weifang, China; 2 Affiliated Hospital of Shandong Second Medical University, Weifang, China; 3 Institute of Clinical Medicine, Peking Union Medical College Hospital, Chinese Academy of Medical Science and Peking Union Medical College, Beijing, China; 4 State Key Laboratory of Complex Severe and Rare Diseases, Beijing, China; 5 Shandong University of Traditional Chinese Medicine Affiliated Hospital, Jinan, China

**Keywords:** self-reported health, cardiovascular diseases, metabolic diseases, multimorbidity, Older adults

## Abstract

**Background:**

While self-reported health (SRH) robustly predicts clinical outcomes, its quantitative association with cardiometabolic multimorbidity (CMM) remains insufficiently characterized, particularly within low- and middle-income countries (LMICs).

**Aims:**

We aimed to quantify the dose-response relationship between SRH and CMM prevalence among older adults in China and to identify key sociodemographic effect modifiers.

**Methods:**

Utilizing cross-sectional data from the 2018 Chinese Longitudinal Healthy Longevity Survey (CLHLS), we analyzed 9,762 participants aged ≥65 years (mean age 83.2 ± 11.3). SRH was categorized as good, neutral, or bad. CMM was defined as the presence of ≥2 conditions among coronary heart disease, stroke, diabetes, hypertension, or dyslipidemia. Multivariable-adjusted logistic regression assessed the SRH-CMM association. Stratified analyses evaluated effect modification by sex, age group, and residence (city/town/rural).

**Results:**

Declining SRH demonstrated a graded association with increased CMM prevalence. Compared to “good” SRH, “bad” SRH was associated with a four-fold elevated CMM risk [adjusted odds ratio (aOR) = 3.992, 95% confidence interval (CI): 3.425–4.652], while “neutral” SRH conferred a two-fold risk elevation (aOR = 2.063, 95% CI: 1.835–2.320). Each one-level deterioration in SRH was associated with more than a doubling of the odds (aOR = 2.009, *p* < 0.001). The association was significantly stronger in males (aOR for bad vs. good = 4.441) than in females (aOR = 3.727), peaked among individuals aged 65–74 years (aOR = 4.785), and attenuated in centenarians (aOR = 3.441). City residents exhibited the highest risk elevation (aOR = 5.326, 95% CI: 3.961–7.163) compared to their rural counterparts (aOR = 3.662, 95% CI: 2.851–4.704; P-interaction = 0.006).

**Conclusions:**

SRH exhibits a strong, independent dose-dependent association with CMM burden in older adults, capturing cumulative biological aging beyond traditional biomarkers. Integrating SRH into clinical risk stratification may optimize preventive interventions for high-risk subgroups, particularly older city males and individuals reporting health deterioration.

## Introduction

Cardiometabolic diseases (CMDs), encompassing hypertension, diabetes, coronary heart disease, stroke, and dyslipidemia, represent major global contributors to morbidity and mortality (1). As life expectancy continues to rise, the prevalence of multimorbidity—defined as the coexistence of two or more chronic conditions—has emerged as the predominant health profile among older adults worldwide (2). In China, rapid demographic aging has intensified this public health challenge, with over 75% of adults aged 60 years and older reporting at least one chronic disease and up to 81.3% experiencing multiple comorbidities (3). Such multimorbidity patterns not only accelerate physiological decline but also amplify healthcare utilization and mortality risks, necessitating innovative strategies to identify high-risk individuals for preventive interventions.

Self-reported health (SRH), a subjective health metric derived from the question “How do you feel about your health condition now?” has emerged as a robust predictor of clinical outcomes across diverse populations (4). Unlike traditional biomarkers, SRH encapsulates multidimensional health perceptions, integrating somatic symptoms, psychological wellbeing, and socioenvironmental contexts into a single, patient-centered indicator (5). Prior research has consistently linked poorer SRH to elevated risks of functional impairment, depression, and all-cause mortality (6). At the same time, longitudinal evidence demonstrates its predictive validity for incident cardiometabolic conditions, including type 2 diabetes in older adult cohorts (7). Despite these associations, the utility of SRH as a marker of cardiometabolic multimorbidity (CMM), a clinical phenotype characterized by synergistic pathophysiology and cumulative organ damage, remains underexplored. This knowledge gap is particularly critical in low- and middle-income countries (LMICs), where demographic transitions and healthcare resource constraints necessitate pragmatic risk-stratification tools.

Existing investigations into SRH-disease relationships have predominantly focused on Western populations or isolated chronic conditions, neglecting the synergistic effects of comorbid CMDs (8, 9). Notably, no large-scale epidemiological study has explicitly examined the association between SRH and CMM in LMICs undergoing rapid aging, limiting the generalizability of findings to resource-limited settings where multimorbidity prevalence is rising sharply. Addressing this gap, the present study leverages the nationally representative Chinese Longitudinal Healthy Longevity Survey (CLHLS) to investigate: (1) the cross-sectional relationship between SRH and CMM prevalence among Chinese adults aged ≥65 years; and (2) the moderating influence of sociodemographic factors—including residence, age, and sex—on this association. By characterizing SRH as a low-cost, patient-reported indicator of CMM risk, this work advances the development of integrated care models tailored to aging populations in LMICs, where scalable solutions for chronic disease management are urgently needed.

## Methods

### Data source

The present study utilized data from the Chinese Longitudinal Healthy Longevity Survey (CLHLS), a nationally representative longitudinal cohort administered jointly by the Center for Healthy Aging and Development Studies at Peking University and the Chinese Academy of Medical Sciences. Initiated in 1998 with follow-up waves conducted biennially through 2018, the CLHLS enrolled adults aged ≥65 years across 23 provinces, autonomous regions, and municipalities, collectively representing ~85% of China's population. Trained interviewers administered in-home assessments using standardized questionnaires capturing sociodemographic characteristics, lifestyle behaviors, and self-reported health metrics. The 2018 wave (*n* = 15,874) served as the analytical basis for this investigation (10).

### Ethics approval

The Institutional Review Board of Peking University (IRB00001052-13074) has approved the ethical review for the CLHLS study. All participants or their authorized representatives have signed written informed consent forms. Participating in the CLHLS study is a voluntary action, and participants have the right to withdraw from the study at any time without suffering any adverse consequences.

### Study population

**Figure 1:**
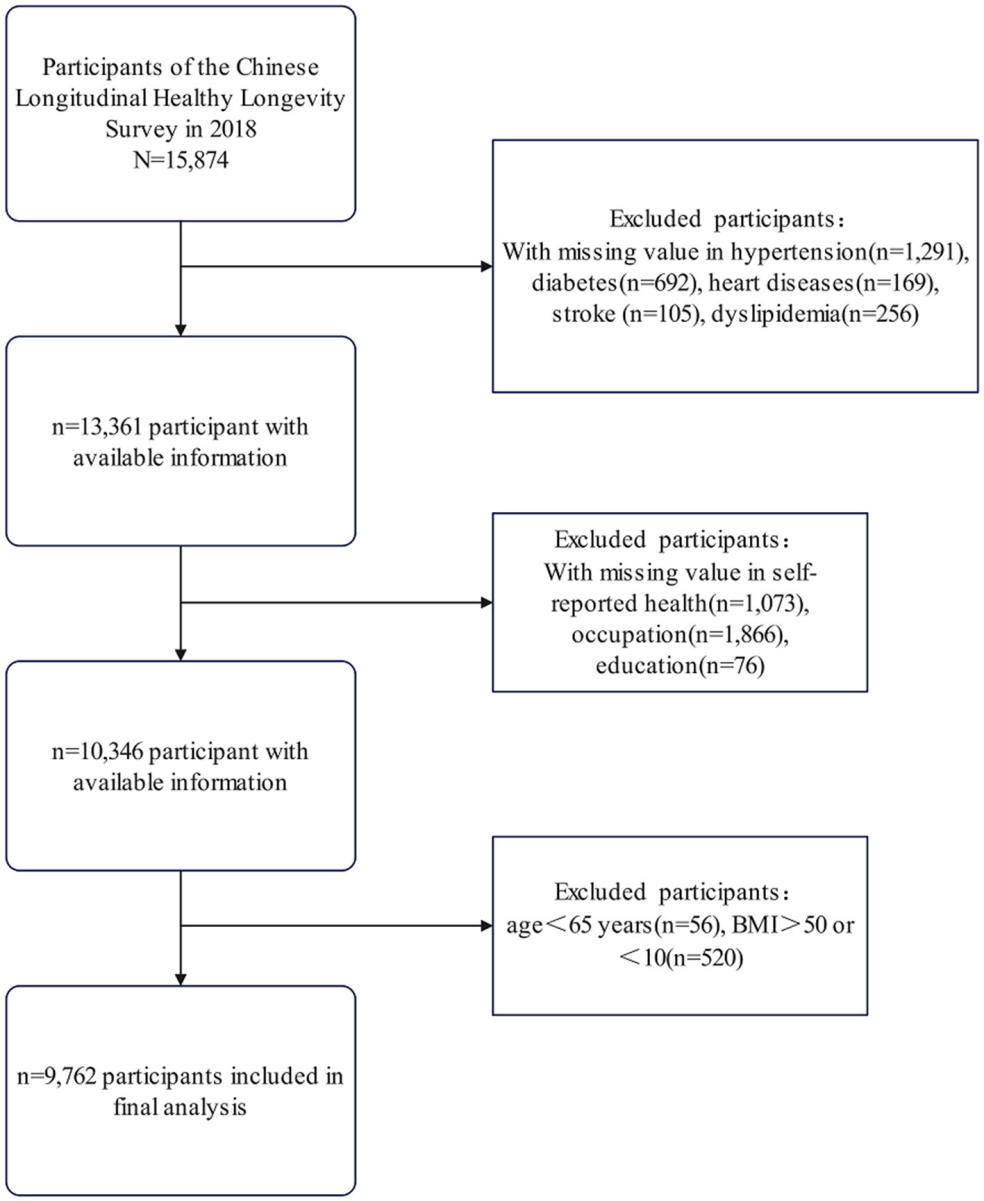
Participant selection flowchart. Flowchart of participant selection from the 2018 Chinese longitudinal healthy longevity survey, showing exclusions due to missing data and criteria, resulting in 9,762 participants included in the final analysis.

This study utilized data from the 2018 wave of the Chinese Longitudinal Healthy Longevity Survey (CLHLS) as the initial sample (*n* = 15,874). The inclusion criterion was participation in the 2018 survey. We then applied the following exclusion criteria sequentially to form the analytical cohort: (1) missing data for primary exposure (self-reported health) or outcome variables (cardiometabolic multimorbidity); (2) covariates with ≥10% missingness (education, occupation); (3) Age =65 years—a criterion consistent with both the age classifications of the World Health Organization (WHO) and the design of the CLHLS cohort (11); and (4) biologically implausible values [body mass index (BMI) < 10 or >50 kg/m^2^]. The final analytic sample comprised 9,762 participants (Figure 1).

### Self-reported health (SRH)

The assessment of SRH is conducted through a single-choice question: “How do you feel about your health condition now?” Responses were recorded on a five-point Likert scale (1 = very good to 5 = very bad). For analysis, SRH was dichotomized into three categories: Good (1–2), Neutral (3), and Bad (4–5), with lower scores indicating better perceived health.

### Cardiometabolic multimorbidity (CMM)

CMM was defined as the simultaneous presence of ≥2 of the following physician-diagnosed conditions: hypertension, diabetes, coronary heart disease, stroke, or dyslipidemia (12). Disease status was self-reported and verified by the question: “Has a doctor ever diagnosed you with [condition]?” Participants responding “don't know” were excluded from CMM calculations. CMM prevalence served as the primary outcome, while individual cardiometabolic conditions were analyzed as secondary endpoints.

### Covariates

A comprehensive set of demographic and health-related variables was incorporated as covariates in the analysis. These included age categories (65–74, 75–84, 85–94, and 95 years and above), sex (male or female), occupation (farmer or non-farmer), place of residence (city, town, or rural), marital status (married and living together or other), body mass index (BMI; < 18.5, 18.5–24, 24–28, >28 kg/m^2^), living arrangement (living with family members, living alone, or residing in an institution), smoking status (yes or no), alcohol consumption status (yes or no), exercise status (yes or no), and educational attainment (illiterate, primary school, middle school or above). Age was categorized to capture non-linear mortality and morbidity gradients across advanced age groups (13, 14). BMI was categorized using Chinese-specific cut-offs for clinical relevance and interpretability (15, 16). Covariates were selected *a priori* based on their established association with SRH and CMM in the literature (17, 18), covering sociodemographic, behavioral, and clinical domains to ensure comprehensive adjustment for potential confounding factors. Details regarding the variables are shown in Table 1.

**Table 1:** Characteristics of the study population (*N* = 9,762).

**Variable**	**Total (*N* = 9,762)**	**Good (*n* = 4,716)**	**Neutral (*n* = 3,743)**	**Bad (*n* = 1,303)**	***p*-Value**
**Age, no. (%)**					
65–74	2,526 (25.8)	1,245 (26.4)	962 (25.7)	319 (24.5)	0.071
75–84	2,678 (27.5)	1,230 (26.1)	1,071 (28.6)	377 (28.9)	
85–94	2,358 (24.2)	1,136 (24.1)	894 (23.9)	328 (25.2)	
≥95	2,200 (22.5)	1,105 (23.4)	816 (21.8)	279 (21.4)	
**Sex, no. (%)**					
Male	4,452 (45.6)	2,250 (47.7)	1,649 (44.1)	553 (42.4)	< 0.001
Female	5,310 (54.4)	2,466 (52.3)	2,094 (55.9)	750 (57.6)	
**Marital status, no. (%)**					
Married	4,271 (43.8)	2,028 (43.0)	1,668 (44.6)	575 (44.1)	0.382
Partnered/single/widowed	5,491 (56.2)	2,688 (57.0)	2,075 (55.4)	728 (55.9)	
**Residence, no. (%)**					
City	2,366 (24.2)	1,186 (25.1)	869 (23.2)	311 (23.9)	0.177
Town	3,298 (33.8)	1,559 (33.1)	1,309 (35.0)	430 (33.0)	
Rural	4,098 (42.0)	1,971 (41.8)	1,565 (41.8)	562 (43.1)	
**Body mass index (BMI), no. (%)**					
< 18.5	1,548 (15.9)	656 (13.9)	609 (16.3)	283 (21.7)	< 0.001
18.5–24	5,143 (52.7)	2,511 (53.2)	1,989 (53.1)	643 (49.3)	
24–28	2,300 (23.5)	1,162 (24.6)	865 (23.1)	273 (21.0)	
>28	771 (7.9)	387 (8.3)	280 (7.5)	104 (8.0)	
**Co-residence, no. (%)**					
Living with house member(s)	7,914 (81.1)	3,839 (81.4)	3,022 (80.7)	1,053 (80.8)	0.935
Living alone	1,562 (16.0)	741 (15.7)	611 (16.4)	210 (16.1)	
In an institution	286 (2.9)	136 (2.9)	110 (2.9)	40 (3.1)	
**Smoking, no. (%)**					
Yes	1,544 (15.8)	823 (17.5)	541 (14.5)	180 (13.8)	< 0.001
No	8,218 (84.2)	3,893 (82.5)	3,202 (85.5)	1,123 (86.2)	
**Drinking, no. (%)**					
Yes	1,498 (15.3)	888 (18.8)	478 (12.8)	133 (10.2)	< 0.001
No	8,264 (84.7)	3,828 (81.2)	3,265 (87.2)	1,170 (89.8)	
**Physical activity, no. (%)**					
Yes	3,326 (34.1)	1,882 (39.9)	1,120 (29.9)	324 (24.9)	< 0.001
No	6,436 (66.9)	2,834 (60.1)	2,623 (70.1)	979 (75.1)	
**Occupation, no. (%)**					
Non-farmer	3,775 (38.7)	1,908 (40.5)	1,380 (36.9)	487 (37.4)	0.002
Farmer	5,987 (61.3)	2,808 (59.5)	2,363 (63.1)	816 (62.6)	
**Education, no. (%)**					
Illiterate	4,416 (45.2)	2,061 (43.7)	1,718 (45.9)	637 (48.9)	0.010
Primary	3,315 (34.0)	1,665 (35.3)	1,245 (33.3)	405 (31.1)	
Middle or higher	2,031 (20.8)	990 (21.0)	780 (20.8)	261 (20.0)	

### Statistical analysis

All analyses were conducted using the SAS 9.4 software. In descriptive statistics, categorical variables were expressed as frequencies (percentages) and compared across SRH categories using χ^2^ tests. Continuous variables were presented as means ± standard deviations (SD) or medians [interquartile ranges] for skewed distributions, with group comparisons conducted via ANOVA or Kruskal-Wallis tests as appropriate. Multivariable logistic regression models quantified associations between SRH and CMM: (1) unadjusted; (2) adjusted for age and sex; and (3) fully adjusted for all covariates (demographic, behavioral, and clinical factors). Adjusted odds ratios (aORs) and 95% confidence intervals (CIs) were reported. Stratified analyses evaluated heterogeneity by sex, residence (city/town/rural), and age group (65–74/75–84/85–94/≥95 years) using likelihood ratio tests for interaction (P-interaction < 0.05). The multiple imputation by chained equations (MICE) method was used to handle the missing covariate data, and the results from the multiply imputed datasets are presented as the primary analysis. This approach maximizes statistical power and minimizes potential bias from missing data. For transparency and to assess the robustness of our findings, a complete-case analysis was also conducted; results are provided in Supplementary Tables S1, S2. Two-tailed *p*-values < 0.05 denote statistical significance.

## Results

### Baseline characteristics

Table 1 presents the baseline characteristics of the study population (*n* = 9,762) stratified by SRH level (good, neutral, bad). Statistically significant differences were observed across all examined domains (*p* < 0.05). While age distributions were comparable across SRH groups (*p* = 0.071), the oldest-old (≥95 years) constituted a larger proportion of the good SRH group (23.4 vs. 21.4% in bad SRH). Males comprised a higher proportion of the good SRH group (47.7%) compared to the neutral (44.1%) and bad SRH groups (42.4%), whereas females constituted a higher proportion in the bad SRH group (57.6%). A gradient correlation was observed in terms of occupation and education. The proportion (40.5%) of non-agricultural occupations was the highest in the group with good SRH, while the prevalence of illiteracy increased progressively with declining SRH levels. Health behavior patterns demonstrated dose-response relationships with SRH, including physical activity participation (39.9% in good SRH vs. 24.9% in bad SRH; *p* < 0.001).

### Sex-stratified disease prevalence patterns

**Figure 2:**
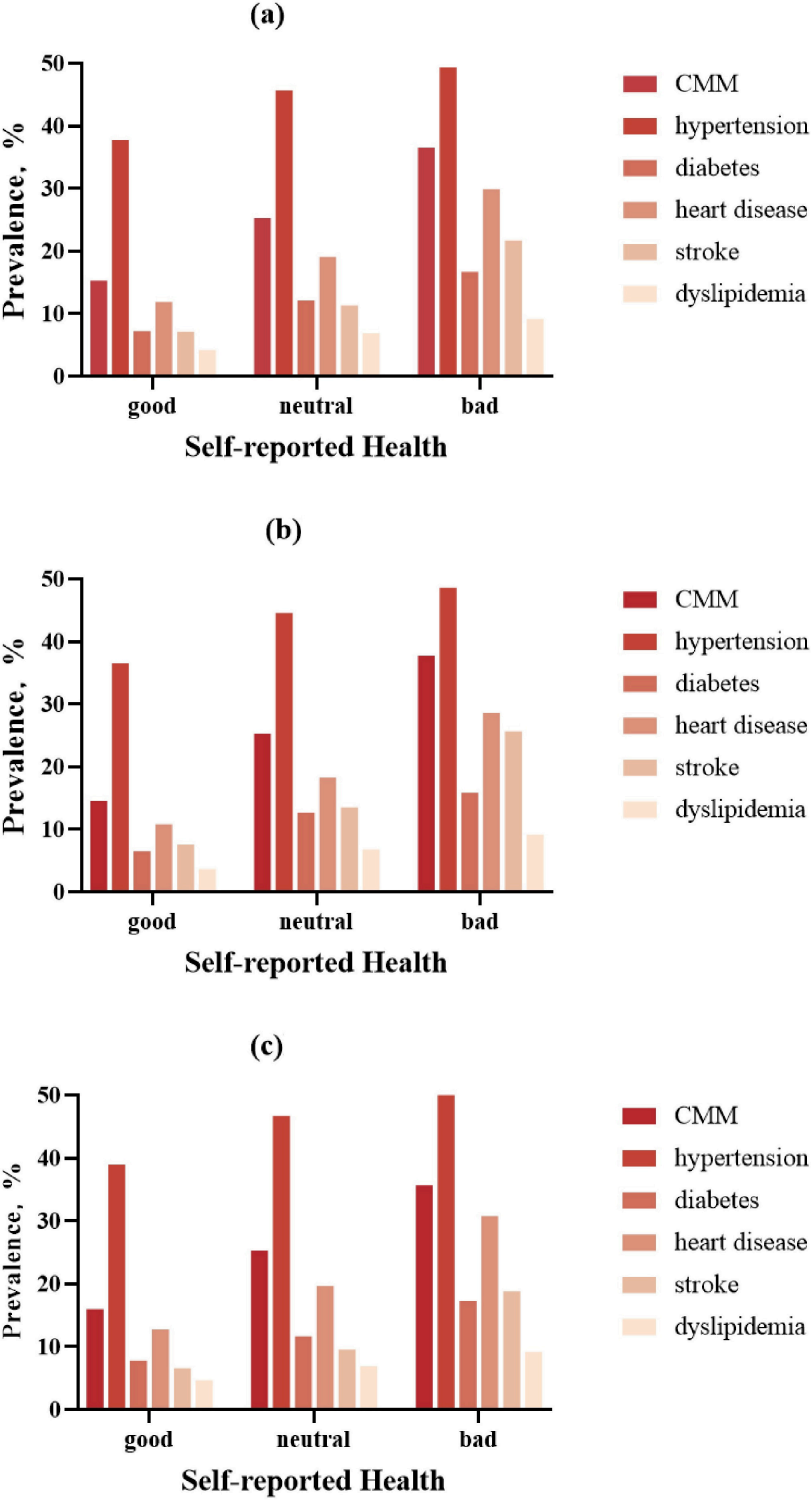
Sex-specific prevalence of cardiometabolic multimorbidity (CMM) and component diseases across SRH categories. Bar charts demonstrating the graded prevalence of: **(a)** overall, **(b)** male, **(c)** female.

Figure 2 illustrates sex-specific prevalence gradients for CMM and its component conditions across SRH categories. In males, CMM prevalence increased from 14.5% (Good SRH) to 37.8% (Bad SRH), with corresponding increases observed for coronary heart disease (10.8 → 28.6%), stroke (7.6 → 25.7%), and diabetes (6.5 → 15.9%). While females exhibited similar directional trends, absolute prevalence differences were attenuated (CMM: 16.0 → 35.7%). Notably, sex-specific prevalence revealed stronger associations in males for most conditions. The female cohort demonstrated a steeper absolute increase in coronary heart disease prevalence when transitioning from neutral to bad SRH (11.1 vs. 10.3% in males).

### Dose-response association between SRH and CMM risk

Table 2 demonstrates a robust, graded relationship between declining SRH and elevated CMM risk. In the fully adjusted model (Model 3), participants reporting bad SRH exhibited 3.992-fold higher odds of CMM compared to those with good SRH (95% CI: 3.425–4.652; *p* < 0.001), with neutral SRH associated with 2.063-fold elevated risk (95% CI: 1.835–2.320). A significant linear trend emerged (*p* < 0.001), with each one-unit decrement in SRH score corresponding to a doubling of CMM odds (aOR = 2.009, 95% CI: 1.865–2.163). Notably, effect sizes strengthened with progressive covariate adjustment: from a crude OR of 3.205 (95% CI: 2.792–3.679) in Model 1 to 3.992 in Model 3. All individual CMD components demonstrated significant dose-response associations (*p* < 0.001 for trend), with particularly strong relationships observed for stroke (bad SRH OR = 3.842; 95% CI: 3.210–4.599) and coronary heart disease (OR = 3.502; 95% CI: 2.992–4.098). Missing data were minimal across all covariates, ranging from 0.8% (living arrangement) to 1.6% (body mass index). The primary analysis utilized multiple imputation (MICE) to address missing values. Results from a complete-case sensitivity analysis were consistent with the primary findings (see Supplementary Tables S1, S2), supporting the robustness of the observed associations.

**Table 2:** The association between self-reported health and cardiometabolic multimorbidity (*N* = 9,762).

**Outcome**	**Events/no**.	**Model 1**		**Model 2** ^**a**^		**Model 3** ^**b**^	
		**OR (95% CI)**	***p*** **-Value**	**OR (95% CI)**	***p*** **-Value**	**OR (95% CI)**	***p*** **-Value**
**Cardiometabolic multimorbidity**							
Self-reported health			< 0.001		< 0.001		< 0.001
Good	720/4,716	Reference		Reference		Reference	
Neutral	947/3,743	1.880 (1.687–2.095)	< 0.001	1.864 (1.670–2.080)	< 0.001	2.063 (1.835–2.320)	< 0.001
Bad	477/1,303	3.205 (2.792–3.679)	< 0.001	3.228 (2.805–3.714)	< 0.001	3.992 (3.425–4.652)	< 0.001
Continuous	2,144/9,762	1.804 (1.687–1.929)	< 0.001	1.807 (1.689–1.934)	< 0.001	2.009 (1.865–2.163)	< 0.001
**Hypertension**							
Self-reported health			< 0.001		< 0.001		< 0.001
Good	1,784/4,716	Reference		Reference		Reference	
Neutral	1,712/3,743	1.385 (1.270–1.512)	< 0.001	1.367 (1.251–1.493)	< 0.001	1.453 (1.325–1.593)	< 0.001
Bad	644/1,303	1.606 (1.419–1.817)	< 0.001	1.583 (1.396–1.794)	< 0.001	1.753 (1.538–1.998)	< 0.001
Continuous	4,140/9,762	1.297 (1.225–1.374)	< 0.001	1.286 (1.213–1.362)	< 0.001	1.356 (1.276–1.441)	< 0.001
**Diabetes**							
Self-reported health			< 0.001		< 0.001		< 0.001
Good	338/4,716	Reference		Reference		Reference	
Neutral	454/3,743	1.788 (1.542–2.073)	< 0.001	1.762 (1.517–2.046)	< 0.001	1.823 (1.560–2.131)	< 0.001
Bad	218/1,303	2.602 (2.168–3.124)	< 0.001	2.595 (2.156–3.125)	< 0.001	2.868 (2.355–3.493)	< 0.001
Continuous	1,010/9,762	1.633 (1.495–1.784)	< 0.001	1.629 (1.489–1.782)	< 0.001	1.710 (1.554–1.882)	< 0.001
**Heart disease**							
Self-reported health			< 0.001		< 0.001		< 0.001
Good	559/4,716	Reference		Reference		Reference	
Neutral	714/3,743	1.753 (1.554–1.977)	< 0.001	1.727 (1.531–1.948)	< 0.001	1.830 (1.614–2.075)	< 0.001
Bad	389/1,303	3.165 (2.730–3.669)	< 0.001	3.115 (2.685–3.614)	< 0.001	3.502 (2.992–4.098)	< 0.001
Continuous	1,662/9,762	1.776 (1.651–1.909)	< 0.001	1.760 (1.636–1.893)	< 0.001	1.866 (1.727–2.015)	< 0.001
**Stroke or cardiovascular disease**							
Self-reported health			< 0.001		< 0.001		< 0.001
Good	333/4,716	Reference		Reference		Reference	
Neutral	423/3,743	1.677 (1.443–1.949)	< 0.001	1.677 (1.441–1.950)	< 0.001	1.700 (1.457–1.984)	< 0.001
Bad	283/1,303	3.652 (3.073–4.339)	< 0.001	3.682 (3.095–4.381)	< 0.001	3.842 (3.210–4.599)	< 0.001
Continuous	1,039/9,762	1.893 (1.735–2.066)	< 0.001	1.900 (1.740–2.075)	< 0.001	1.939 (1.770–2.124)	< 0.001
**Dyslipidemia**							
Self-reported health			< 0.001		< 0.001		< 0.001
Good	198/4,716	Reference		Reference		Reference	
Neutral	257/3,743	1.682 (1.390–2.035)	< 0.001	1.647 (1.359–1.996)	< 0.001	1.737 (1.421–2.123)	< 0.001
Bad	120/1,303	2.315 (1.829–2.930)	< 0.001	2.284 (1.800–2.898)	< 0.001	2.649 (2.055–3.415)	< 0.001
Continuous	575/9,762	1.540 (1.375–1.725)	< 0.001	1.527 (1.361–1.714)	< 0.001	1.642 (1.451–1.857)	< 0.001

^a^Adjusted for sex and age.

^b^Based on model 2, additionally adjusted for place of residence, educational attainment, living arrangement, marital status, occupation, smoking, drinking, exercise, and body mass index.

### Demographic modifiers of the SRH-CMM association

**Figure 3:**
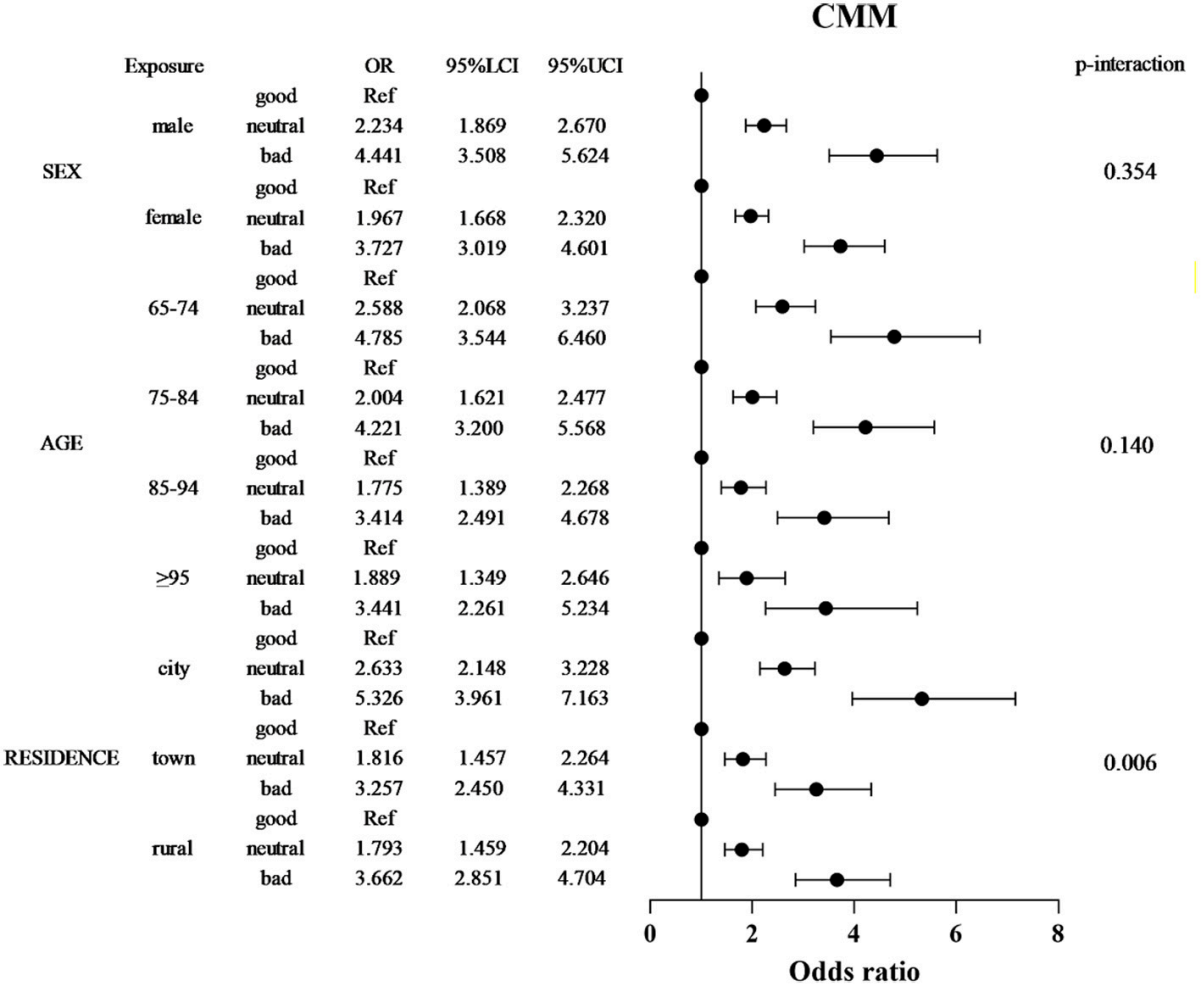
Stratified forest plot of SRH-CMM associations by sex, age, and residence. Forest plot depicting adjusted odds ratios (ORs) and 95% confidence intervals (CIs) for the relationship between self-reported health (SRH) and CMM risk, stratified by sex, age group, and residence. Apart from the stratification factors, the fully adjusted model (Model 3) is based on multiply imputed data and controls for sex, age, place of residence, educational attainment, living arrangement, marital status, occupation, smoking, drinking, exercise, and body mass index. Forest plots for individual cardiometabolic conditions are shown in Supplementary Figures S1–S5.

Stratified analyses revealed significant effect modification by sociodemographic factors (Figure 3). Sex differences were pronounced, with males exhibiting stronger SRH-CMM associations (bad SRH OR = 4.441, 95% CI: 3.508–5.624) compared to females (OR = 3.727, 95% CI: 3.019–4.601; P-interaction = 0.354). Age-specific effects demonstrated attenuation with advancing age: the strongest association occurred in the 65–74 age group (bad SRH OR = 4.785, 95% CI: 3.544–6.460), declining to 3.441 (95% CI: 2.261–5.234) in those ≥95 years (P for trend < 0.001). City residence amplified risk estimates, with bad SRH associated with 5.326-fold higher CMM odds in city dwellers (95% CI: 3.961–7.163) compared to 3.662-fold in rural residents (95% CI: 2.851–4.704; P-interaction = 0.006). Notably, neutral SRH conferred elevated risk only in city participants (OR = 2.633; 95% CI: 2.148–3.228). The stratified associations between SRH and CMM across key demographic subgroups are presented in Figure 3. Furthermore, forest plots for the associations between SRH and each individual cardiometabolic condition (hypertension, diabetes, coronary heart disease, stroke, dyslipidemia) are provided in Supplementary Figures S1–S5. These analyses reveal patterns generally consistent with the overall CMM findings.

## Discussions

The operational definitions of SRH and CMM adopted in this study warrant specific discussion to situate our findings within the existing literature. Our use of a single-item, five-level SRH measure is consistent with large-scale aging surveys such as the Health and Retirement Study (HRS) and the Survey of Health, Aging and Retirement in Europe (SHARE) (4, 19). This approach prioritizes comparability across studies and captures a global perception of health. It should be noted, however, that this differs from instruments designed to evaluate disease-specific impacts, such as the EQ-5D; thus, our SRH measure reflects a holistic appraisal that may be influenced by health factors beyond cardiometabolic conditions. For CMM, we defined the outcome as the co-occurrence of at least two conditions from a focused set of five common cardiometabolic diseases: hypertension, diabetes, coronary heart disease, stroke, and dyslipidemia. This clinically meaningful threshold and disease combination are widely employed in epidemiological research (12, 20). Nevertheless, some studies employ broader definitions that include conditions like heart failure or chronic kidney disease. While our definition enhances specificity for the cardiometabolic domain, it may underestimate the full multimorbidity burden compared to these broader approaches. These definitional choices support the comparability of our findings with a substantial body of research while clarifying the specific clinical phenotype under investigation.

### Multimorbidity as a biopsychosocial construct

This study establishes a robust, dose-dependent association between SRH and CMM in older Chinese, demonstrating that each one-unit decline in SRH corresponds to a doubling of CMM risk. While prior research has linked bad SRH to individual CMDs (21, 22), population-based investigations reinforce this concept: a Korean community study found cardiovascular disease, diabetes, and hypertension significantly increased poor SRH odds (23), while research in Canadian First Nations populations demonstrated diabetes's strong association with diminished SRH (24). Our findings extend this paradigm by positioning SRH as a holistic indicator of cumulative biological burden. The four-fold elevated CMM risk in individuals reporting “bad” SRH exceeds previous reports from European cohorts (e.g., OR = 2.820 for diabetes-specific associations) (25). It aligns with evidence from type 2 diabetes cohorts where multimorbidity (≥3 conditions) increased poor SRH odds tenfold (26). This suggests SRH may capture synergistic pathophysiological interactions inherent to multimorbidity. This integrative capacity is further evidenced in diabetic peripheral neuropathy patients, where comorbid cardiovascular/renal disease is independently associated with lower SRH ratings (27), and hypertension studies confirming SRH's predictive value for major cardiovascular events (28). This aligns with the allostatic load framework, where SRH integrates subclinical vascular dysfunction, chronic inflammation, and metabolic dysregulation into a single, patient-centered metric.

### Sex-specific pathways linking SRH to CMM

This sex-stratified analysis revealed a robust dose-dependent increase in CMM prevalence across declining SRH. In males, prevalence rose from 14.5% (“good” SRH) to 37.8% (“bad” SRH), while females exhibited a significant though attenuated gradient (16.0 to 35.7%). This pattern likely reflects accruing subclinical vascular pathology—including microvascular injury, chronic inflammation, and autonomic dysfunction—accelerating cardiometabolic decompensation (29, 30). Notably, males demonstrated consistently higher stroke prevalence across SRH strata, aligning with evidence of androgen-mediated endothelial dysfunction and prothrombotic states that amplify cerebrovascular risk (31). Conversely, females exhibited exaggerated SRH-associated increases in coronary heart disease (30.8 vs. 28.6% in males with “bad” SRH) and diabetes (17.3 vs. 15.9% in males with “bad” SRH). This steeper gradient likely reflects postmenopausal estrogen decline, which disrupts metabolic homeostasis by promoting visceral adiposity, insulin resistance, and coronary microvascular dysfunction—pathological processes often undetected by conventional angiography yet clinically significant in symptom burden and health perception (32, 33).

Clinically, SRH serves as a pragmatic primary care risk stratification tool. A single-item SRH measure could identify high-risk individuals for targeted interventions, including lifestyle modification and polypharmacy optimization, particularly among older men and postmenopausal women. Integrating SRH into clinical algorithms may enhance early detection of multisystem dysregulation, offering a low-cost complement to traditional biomarker-based approaches in resource-constrained settings.

### Demographic contextualization of risk

Stratified analyses reveal context-specific modulation of the SRH-CMM relationship by sex, age, and residence, reflecting intersecting biological and environmental determinants of health perception. These modifiers position SRH as a dynamic biomarker of cumulative pathophysiological stress, integrating individual susceptibility with systemic exposures.

The pronounced SRH-CMM association in males reflects androgen-driven vascular pathophysiology. Testosterone exacerbates platelet hyperreactivity and endothelial dysfunction, accelerating subclinical atherosclerosis in metabolically compromised males (34, 35). Concurrently, male-pattern visceral adiposity promotes secretion of IL-6 and PAI-1, creating a feed-forward loop where metabolic insults disproportionately degrade perceived health (36).

Age-related attenuation of the SRH-CMM gradient highlights competing physiological processes. Younger older adults retain sufficient functional reserve to manifest SRH sensitivity to emerging dysreallostatic load, whereas extreme-age survivors exhibit selective resilience to fatal CMM and competing comorbidities (37). This “diagnostic saturation” in centenarians reduces SRH specificity, masking underlying cardiometabolic burden through survivorship bias (38).

City residence amplified CMM risk (OR = 5.326 for “bad” SRH vs. 3.662 in rural areas), suggesting environmental cardiometabolic toxicity. Chronic exposure to airborne particulates and psychosocial stressors may induce systemic inflammation, endothelial activation, and lipid peroxidation, thereby accelerating atherosclerosis and hypertension pathogenesis (39). These city penalties exceed rural-city disparities in traditional risk factors, implicating environmental stress as a mediator of SRH deterioration.

### Theoretical and clinical implications

These findings position SRH as a low-cost, integrative biomarker of multisystem dysregulation, with particular utility in resource-limited settings. The four-fold CMM risk elevation in “bad” SRH exceeds the predictive power of traditional risk scores, suggesting SRH could enhance risk stratification in primary care. Clinically, a single-item SRH assessment may identify high-risk individuals for targeted interventions (e.g., polypharmacy optimization, lifestyle counseling), particularly among older men and city dwellers. The graded relationship across all CMM components underscores SRH's value in capturing cumulative biological age, independent of chronological age.

### Limitations and future directions

This study also has several limitations that should be considered. First, as rightly noted, SRH is a broad, subjective measure of general health perception and was not designed to specifically capture the impact of cardiometabolic conditions. As a holistic indicator, an individual's SRH rating is likely influenced by a wide range of factors beyond the CMM definition used in this study, including other physical comorbidities (e.g., arthritis, chronic respiratory diseases), mental health status (e.g., depression), and sensory or functional impairments. While we adjusted for several key confounders, the possibility of residual confounding by these unmeasured health issues cannot be ruled out. This inherent characteristic of SRH may partly explain its strong association with CMM, as it potentially reflects the cumulative burden of all health deficits, not solely cardiometabolic ones. Therefore, the observed association should be interpreted as SRH capturing a general state of biological aging and overall disease burden, with CMM being a major, but not exclusive, component.

In addition to the limitations of the measure itself, this study has methodological limitations. While it benefits from a nationally representative sample and comprehensive confounder adjustment, its cross-sectional design precludes causal inference. Longitudinal analyses are required to establish SRH's predictive validity for incident CMM and to disentangle temporal relationships (e.g., whether declining SRH precedes or results from multimorbidity). Additionally, cultural variations in health perception may limit generalizability to other populations. Future work should explore biological mediators of the SRH-CMM link, such as inflammatory markers or cellular aging indices, to better elucidate the underlying mechanisms.

## Conclusions

This study provides the first large-scale evidence of a graded SRH-CMM relationship in an older adult population, demonstrating that SRH captures a cumulative biological burden beyond traditional biomarkers. Integrating this metric into clinical algorithms could enhance risk stratification in LMIC health systems without reliance on costly diagnostics. These findings advocate for SRH's inclusion in global aging research, with critical relevance to China's rapidly aging population and escalating multimorbidity burden.

## Data Availability

Publicly available datasets were analyzed in this study. The data can be accessed on the Open Research Data Platform of Peking University: https://opendata.pku.edu.cn/dataverse/CHADS (Reference Number: IRB00001052-13074). To access these data, registration and approval through the official platform are required.
