# Supplementary material for: Self-reported health as a predictor of cardiometabolic multimorbidity in Chinese older adults: a national cross-sectional study
Source: Front Public Health. 2025 Nov 14;13:1691960. doi: 10.3389/fpubh.2025.1691960 (PMC12660099; doi:10.3389/fpubh.2025.1691960)
Supplement: Supplementary file 1 [file Table_1.docx]

***Supplementary Material***

**Table S1** Characteristics of the study population before multiple imputation.

| Variable | | Total | Good | Neutral | Bad | P-value |
| --- | --- | --- | --- | --- | --- | --- |
| **Age, No. (%)** | | | | | | 0.071 |
|  | 65-74 | 2526(25.9) | 1245(26.4) | 962(25.7) | 319(24.5) |  |
|  | 75-84 | 2678(27.4) | 1230(26.1) | 1071(28.6) | 377(28.9) |  |
|  | 85-94 | 2358(24.2) | 1136(24.1) | 894(23.9) | 328(25.2) |  |
|  | ≥95 | 2200(22.5) | 1105(23.4) | 816(21.8) | 279(21.4) |  |
| **Sex, No. (%)** | | | | | | ＜0.001 |
|  | male | 4452(45.6) | 2250(47.7) | 1649(44.1) | 553(42.4) |  |
|  | female | 5310(54.4) | 2466(52.3) | 2094(55.9) | 750(57.6) |  |
| **Marital status, No. (%)^a^** | | | | | | 0.317 |
|  | married | 4233(43.8) | 2011(43.0) | 1653(44.6) | 569(44.1) |  |
|  | partnered/single/widowed | 5440(56.2) | 2667(57.0) | 2052(55.4) | 721(55.9) |  |
| **Residence, No. (%)** | | | | | | 0.177 |
|  | city | 2366(24.2) | 1186(25.1) | 869(23.2) | 311(23.9) |  |
|  | town | 3298(33.8) | 1559(33.1) | 1309(35.0) | 430(33.0) |  |
|  | rural | 4098(42.0) | 1971(41.8) | 1565(41.8) | 562(43.1) |  |
| **Body Mass Index(BMI), No. (%)^a^** | | | | | | ＜0.001 |
|  | ＜18.5 | 1528(15.9) | 644(13.9) | 602(16.4) | 282(21.9) |  |
|  | 18.5-24 | 5062(52.7) | 2471(53.3) | 1955(53.1) | 636(49.3) |  |
|  | 24-28 | 2255(23.5) | 1137(24.5) | 850(23.1) | 268(20.8) |  |
|  | ＞28 | 756(7.9) | 380(8.3) | 273(7.4) | 103(8.0) |  |
| **Co-residence, No. (%)^a^** | | | | | | 0.925 |
|  | living with house member(s) | 7851(81.1) | 3818(81.4) | 2989(80.7) | 1044(80.8) |  |
|  | living alone | 1552(16.0) | 737(15.7) | 607(16.4) | 208(16.1) |  |
|  | in an institution | 283(2.9) | 135(2.9) | 108(2.9) | 40(3.1) |  |
| **Smoking, No. (%)^a^** | | | | | | ＜0.001 |
|  | yes | 1535(15.9) | 818(17.5) | 538(14.5) | 179(13.9) |  |
|  | no | 8134(84.1) | 3849(82.5) | 3172(85.5) | 1113(86.1) |  |
| **Drinking, No. (%)^a^** | | | | | | ＜0.001 |
|  | yes | 1482(15.4) | 880(18.9) | 471(12.8) | 131(10.1) |  |
|  | no | 8137(84.6) | 3770(81.1) | 3207(87.2) | 1160(89.9) |  |
| **Physical activity, No. (%)^a^** | | | | | | ＜0.001 |
|  | yes | 3291(34.1) | 1865(40.1) | 1105(29.9) | 321(24.8) |  |
|  | no | 6351(65.9) | 2791(59.9) | 2588(70.1) | 972(75.2) |  |
| **Occupation, No. (%)** | | | | | | 0.002 |
|  | non-farmer | 3775(38.7) | 1908(40.5) | 1380(36.9) | 487(37.4) |  |
|  | farmer | 5987(61.3) | 2808(59.5) | 2363(63.1) | 816(62.6) |  |
| **Education, No. (%)** | | | | | | 0.010 |
|  | Illiterate | 4416(45.2) | 2061(43.7) | 1718(45.9) | 637(48.9) |  |
|  | primary | 3315(34.0) | 1665(35.3) | 1245(33.3) | 405(31.1) |  |
|  | middle or higher | 2031(20.8) | 990(21.0) | 780(20.8) | 261(20.0) |  |

^a^ Missing data: 89 participants for marital status (0.9%), 161 for body mass index (1.6%), 76 for co-residence (0.8%), 93 for smoking (1.0%), 143 for drinking (1.5%), 120 for physical activity (1.2%).

**Table S2** The association between self-reported health and cardiometabolic multimorbidity before multiple imputation.

| Outcome | | Events/No. | Model 1 |  | Model 2^a^ |  | Model 3^b^ |  |
| --- | --- | --- | --- | --- | --- | --- | --- | --- |
|  | |  | OR(95%CI) | P-value | OR(95%CI) | P-value | OR(95%CI) | P-value |
| **Cardiometabolic multimorbidity** | | | | | | | | |
| Self-reported health | |  |  | ＜0.001 |  | ＜0.001 |  | ＜0.001 |
|  | good | 720/4716 | Reference |  | Reference |  | Reference |  |
|  | neutral | 947/3743 | 1.880(1.687-2.095) | ＜0.001 | 1.864(1.670-2.080) | ＜0.001 | 2.053(1.818-2.319) | ＜0.001 |
|  | bad | 477/1303 | 3.205(2.792-3.679) | ＜0.001 | 3.228(2.805-3.714) | ＜0.001 | 4.049(3.459-4.740) | ＜0.001 |
| Continuous | | 2144/9762 | 1.804(1.687-1.929) | ＜0.001 | 1.807(1.689-1.934) | ＜0.001 | 2.019(1.870-2.179) | ＜0.001 |
| **Hypertension** | | | | | | | | |
| Self-reported health | |  |  | ＜0.001 |  | ＜0.001 |  | ＜0.001 |
|  | good | 1784/4716 | Reference |  | Reference |  | Reference |  |
|  | neutral | 1712/3743 | 1.385(1.270-1.512) | ＜0.001 | 1.367(1.251-1.493) | ＜0.001 | 1.457(1.324-1.602) | ＜0.001 |
|  | bad | 644/1303 | 1.606(1.419-1.817) | ＜0.001 | 1.583(1.396-1.794) | ＜0.001 | 1.751(1.530-2.004) | ＜0.001 |
| Continuous | | 4140/9762 | 1.297(1.225-1.374) | ＜0.001 | 1.286(1.213-1.362) | ＜0.001 | 1.356(1.274-1.444) | ＜0.001 |
| **Diabetes** | | | | | | | | |
| Self-reported health | |  |  | ＜0.001 |  | ＜0.001 |  | ＜0.001 |
|  | good | 338/4716 | Reference |  | Reference |  | Reference |  |
|  | neutral | 454/3743 | 1.788(1.542-2.073) | ＜0.001 | 1.762(1.517-2.046) | ＜0.001 | 1.798(1.529-2.115) | ＜0.001 |
|  | bad | 218/1303 | 2.602(2.168-3.124) | ＜0.001 | 2.595(2.156-3.125) | ＜0.001 | 2.936(2.397-3.596) | ＜0.001 |
| Continuous | | 1010/9762 | 1.633(1.495-1.784) | ＜0.001 | 1.629(1.489-1.782) | ＜0.001 | 1.724(1.562-1.904) | ＜0.001 |
| **Heart disease** | | | | | | | | |
| Self-reported health | |  |  | ＜0.001 |  | ＜0.001 |  | ＜0.001 |
|  | good | 559/4716 | Reference |  | Reference |  | Reference |  |
|  | neutral | 714/3743 | 1.753(1.554-1.977) | ＜0.001 | 1.727(1.531-1.948) | ＜0.001 | 1.810(1.590-2.060) | ＜0.001 |
|  | bad | 389/1303 | 3.165(2.730-3.669) | ＜0.001 | 3.115(2.685-3.614) | ＜0.001 | 3.531(3.004-4.149) | ＜0.001 |
| Continuous | | 1662/9762 | 1.776(1.651-1.909) | ＜0.001 | 1.760(1.636-1.893) | ＜0.001 | 1.870(1.727-2.024) | ＜0.001 |
| **Stroke or cardiovascular disease** | | | | | | | | |
| Self-reported health | |  |  | ＜0.001 |  | ＜0.001 |  | ＜0.001 |
|  | good | 333/4716 | Reference |  | Reference |  | Reference |  |
|  | neutral | 423/3743 | 1.677(1.443-1.949) | ＜0.001 | 1.677(1.441-1.950) | ＜0.001 | 1.701(1.451-1.996) | ＜0.001 |
|  | bad | 283/1303 | 3.652(3.073-4.339) | ＜0.001 | 3.682(3.095-4.381) | ＜0.001 | 3.879(3.224-4.667) | ＜0.001 |
| Continuous | | 1039/9762 | 1.893(1.735-2.066) | ＜0.001 | 1.900(1.740-2.075) | ＜0.001 | 1.949(1.775-2.140) | ＜0.001 |
| **Dyslipidemia** | | | | | | | | |
| Self-reported health | |  |  | ＜0.001 |  | ＜0.001 |  | ＜0.001 |
|  | good | 198/4716 | Reference |  | Reference |  | Reference |  |
|  | neutral | 257/3743 | 1.682(1.390-2.035) | ＜0.001 | 1.647(1.359-1.996) | ＜0.001 | 1.810(1.470-2.229) | ＜0.001 |
|  | bad | 120/1303 | 2.315(1.829-2.930) | ＜0.001 | 2.284(1.800-2.898) | ＜0.001 | 2.665(2.048-3.467) | ＜0.001 |
| Continuous | | 575/9762 | 1.540(1.375-1.725) | ＜0.001 | 1.527(1.361-1.714) | ＜0.001 | 1.655(1.457-1.879) | ＜0.001 |

^a^ Adjusted for sex and age.

^b^ Based on model 2, additionally adjusted for place of residence, educational attainment, living arrangement, marital status, occupation, smoking, drinking, exercise, and body mass index.


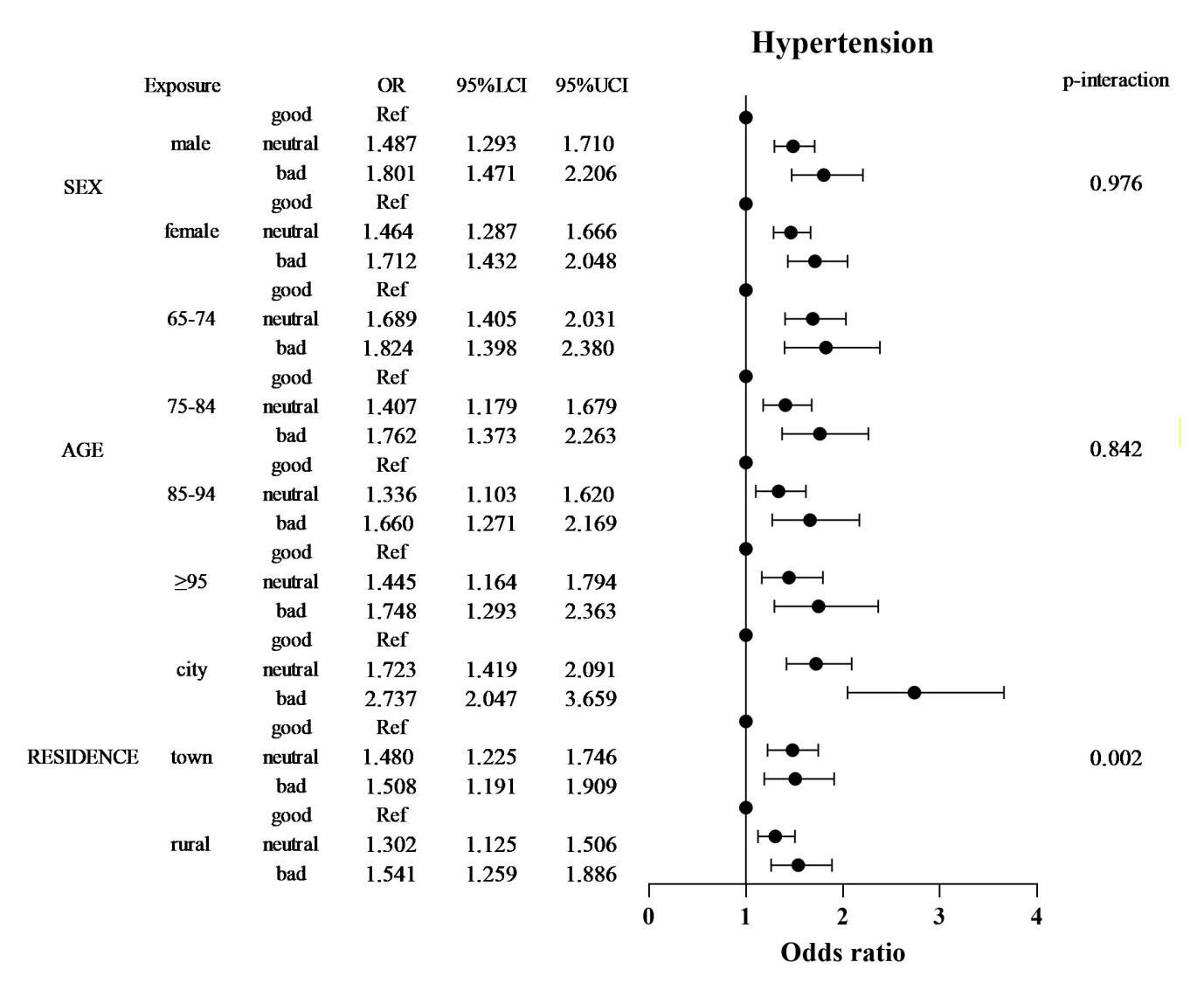
**Figure S1** Stratified forest plot of SRH-Hypertension associations by sex, age, and residence.

**
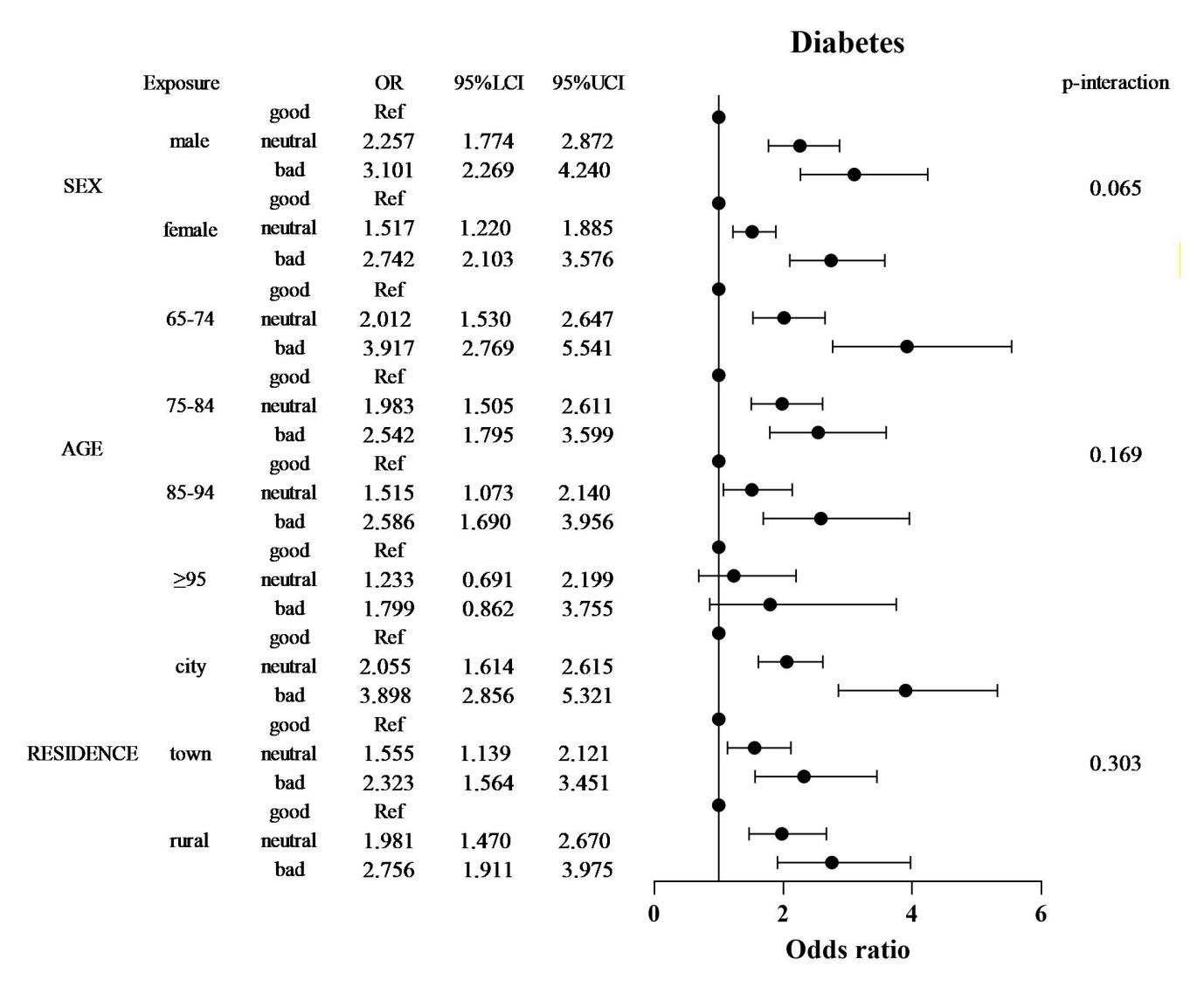
Figure S2** Stratified forest plot of SRH-Diabetes associations by sex, age, and residence.

**
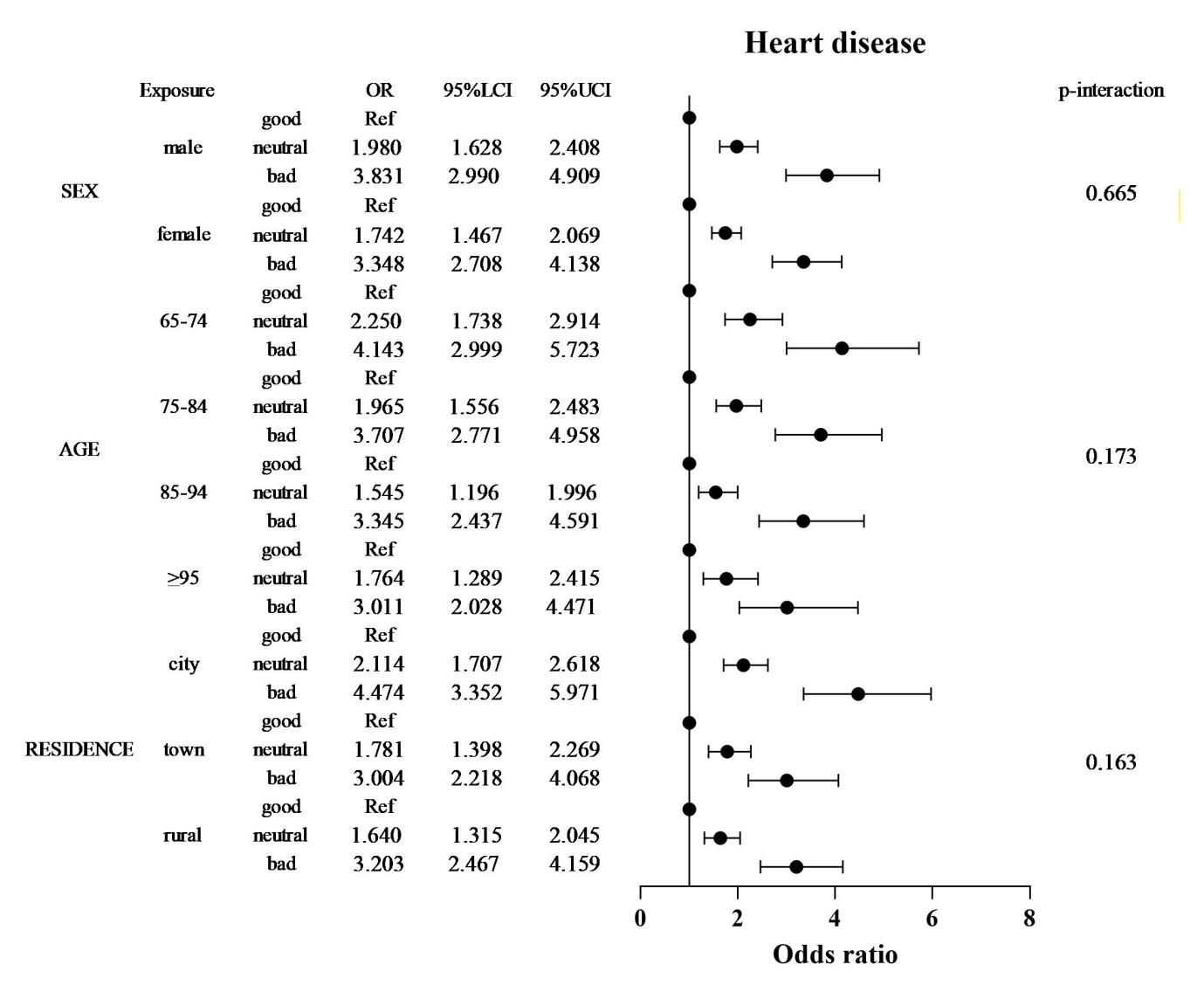
Figure S3** Stratified forest plot of SRH-Heart disease associations by sex, age, and residence.

**
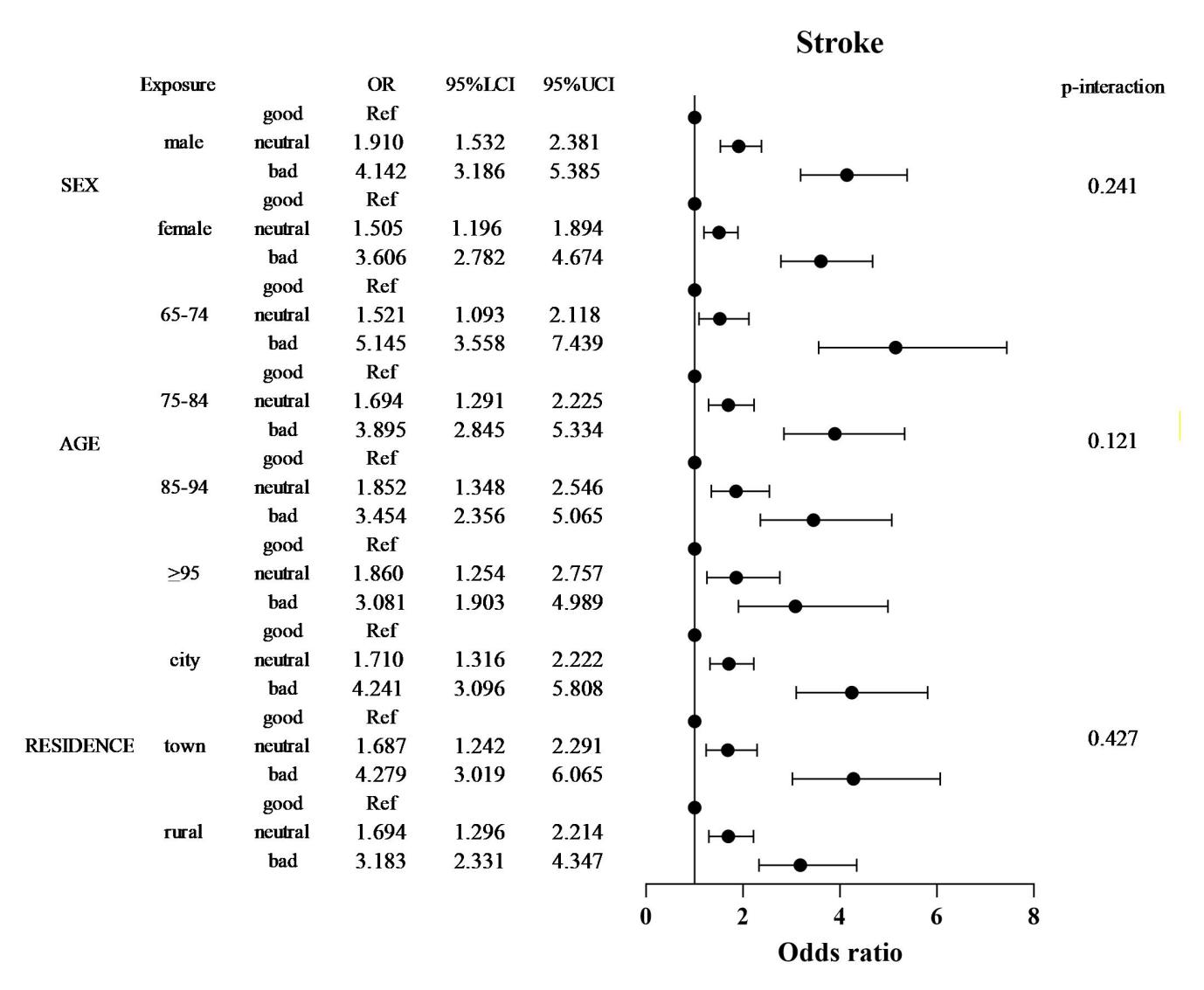
Figure S4** Stratified forest plot of SRH-Stroke associations by sex, age, and residence.

**
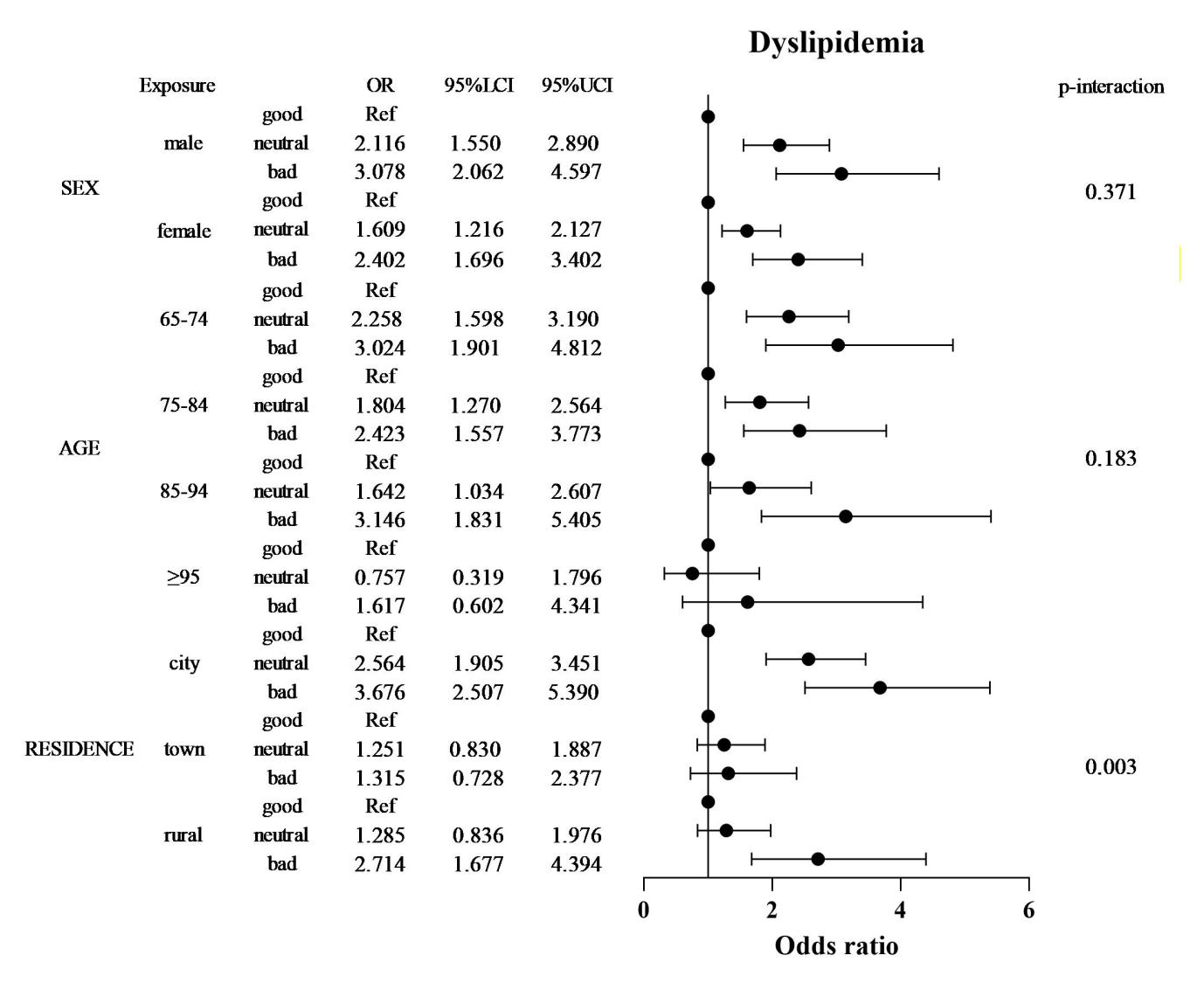
Figure S5** Stratified forest plot of SRH-Dyslipidemia associations by sex, age, and residence.
